# Amino Acid Profiles in Term and Preterm Human Milk through Lactation: A Systematic Review

**DOI:** 10.3390/nu5124800

**Published:** 2013-11-26

**Authors:** Zhiying Zhang, Alicia S. Adelman, Deshanie Rai, Julia Boettcher, Bo Lőnnerdal

**Affiliations:** 1 Department of Kinesiology and Community Health, University of Illinois at Urbana-Champaign, Urbana, IL 61801, USA; E-Mail: zzhang26@illinois.edu; 2 Mead Johnson Pediatric Nutrition Institute, Evansville, IN 47721, USA; E-Mails: adelman.alicia@gmail.com (A.S.A.); deshanierai@gmail.com (D.R.); 3 Department of Nutrition, University of California, Davis, CA 95616, USA; E-Mail: bllonnerdal@ucdavis.edu

**Keywords:** protein, non-protein nitrogen, breast milk, infant nutrition

## Abstract

Amino acid profile is a key aspect of human milk (HM) protein quality. We report a systematic review of total amino acid (TAA) and free amino acid (FAA) profiles, in term and preterm HM derived from 13 and 19 countries, respectively. Of the 83 studies that were critically reviewed, 26 studies with 3774 subjects were summarized for TAA profiles, while 22 studies with 4747 subjects were reviewed for FAA. Effects of gestational age, lactation stage, and geographical region were analyzed by Analysis of Variance. Data on total nitrogen (TN) and TAA composition revealed general inter-study consistency, whereas FAA concentrations varied among studies. TN and all TAA declined in the first two months of lactation and then remained relatively unchanged. In contrast, the FAA glutamic acid and glutamine increased, peaked around three to six months, and then declined. Some significant differences were observed for TAA and FAA, based on gestational age and region. Most regional TAA and FAA data were derived from Asia and Europe, while information from Africa was scant. This systematic review represents a useful evaluation of the amino acid composition of human milk, which is valuable for the assessment of protein quality of breast milk substitutes.

## 1. Introduction

Protein quality and quantity are key aspects of the nutritional value of infant feedings. Although several factors influence protein quality, the amino acid profile of the feeding is well recognized and documented as a contributor to overall protein quality. The protein composition of infant feedings may be evaluated with an amino acid score that is based on human milk amino acid composition [1].

Many of the available references on the amino acid composition of human milk provide values in quantities of total protein or total nitrogen without considering the differences between crude protein derived from total nitrogen and true protein from protein nitrogen. Total amino acids (TAA) are comprised of amino acids contributing to both protein nitrogen (protein-bound amino acids) and non-protein nitrogen (NPN) [2]. A large proportion (around 20%–25%) of total nitrogen in human milk is non-protein nitrogen; free amino acids (FAA) account for 8%–22% of NPN and 5%–10% of TAA [3,4,5,6]. Taurine, glutamic acid, and glutamine are the most abundant free amino acids in human milk, with glutamic acid and glutamine comprising nearly 50% of total free amino acids [7,8,9,10]. Free amino acids contribute to the body’s utilizable nitrogen, are credited for the initial change in plasma free amino acids following a feed, and are more readily absorbed than protein-derived amino acids [5,11]. Increasing evidence suggests that free amino acids may play an important role in early postnatal development, yet their full biological significance has not been fully defined [5,12].

Advances in infant nutrition composition and functionality often begin with a more thorough understanding of human milk. We know of no single comprehensive systematic review of human milk amino acid profiles available in peer-reviewed literature that can serve as a reference for amino acid composition of preterm and term infant feedings. Although preterm human milk is not considered nutritionally adequate to support the intrauterine growth and development of preterm infants [13], human milk offers several physiological and psychological benefits, and experts recommend fortified human milk for preterm infants [14]. Therefore, an understanding of preterm human milk composition is an important step in providing optimal nutrition to this vulnerable population.

This systematic review considered both total and free amino acid profiles in term and preterm breast milk, from mothers living in various geographic locations, from all available literature worldwide. Our goal was to report human milk amino acid concentration from women around the world and throughout the course of lactation, to determine the effects of gestational age, geography, and stage of lactation on the concentration of total and free amino acids.

## 2. Experimental Section

### 2.1. Data Origin

A search of the literature using PubMed, Scopus, EMBASE, Google Scholar, and ProQuest Dissertations & Theses (PQDT)‎ was performed with the keywords “breast milk” or “human milk” and “amino acid”. The most recent search was conducted in February 2010. We also reviewed reference lists to identify any articles not found using online methods. Some reports were translated into English as needed. All data were obtained from original reports.

Data pertaining to the amino acid composition in human milk were categorized by gestational age, stage of lactation, and country. A summary was prepared using the means reported in the literature. The countries were grouped into the wide geographical regions of Asia/Pacific (AP), Europe (EU), North America (NA), and Africa (AF). If gestational age was not specified (*i.e.*, preterm or term), the article was included and categorized as not-specified (NS). Stages of lactation were categorized as colostrum (0–5 days), transitional (6–20 days), and mature (≥21 days).

As there were a large number of mature milk studies, mature milk was further classified into subgroups based on the lactation day. Many studies provided the lactation ranges of pooled milk samples rather than a specific day; these studies were grouped based on their average lactation day. Compared to TAA, fewer studies were found on the FAA profile of mature human milk. Therefore, three subgroups of mature milk (MT) were identified for TAA studies, whereas only two subgroups were named for FAA. Subgroup labels indicate the last lactation day included in that subgroup: MT2mo (21–58 days), MT4mo (59–135 days), and MT18mo (136–540 days) for TAA and MT2mo (21–60 days) and MT > 2 mo (≥61 days) for FAA.

### 2.2. Inclusion Criteria

To be included in this review, the following criteria had to be met. For term human milk, data had to be from studies of “normal” or “healthy” mothers who delivered healthy term infants. Additionally, these mothers had to be consuming free-living diets; data from mothers consuming special diets were excluded. For preterm human milk, data had to be from studies of “normal” or “healthy” mothers who delivered an appropriate for gestational age preterm infant. Due to the paucity of available data on preterm infants, no study was excluded based on infant health or the lack of information thereof. Sufficient information on milk sampling, including stage of lactation, units used to express amino acid concentration, and geographic location also had to be stated. Data from only one mother, means derived from a collection of studies, broad stage of lactation (*i.e.*, mean of all or most stages of lactation), or duplicated representations of data, were excluded. Milk could be obtained with mechanical, electrical, and hand pumps or by manual expression; samples were transported and stored in either liquid or freeze-dried form; defatted or whole milk was used for hydrolysis. Other variables such as age, ethnicity, body weight, socioeconomic status, and season were not considered. Milk samples were from complete 24 h collections, or at least the entire amount of milk from one or both breasts at a feeding, or pooled or banked milk. Studies that used ion exchange chromatography analytical method or specifically indicating using an automatic amino acid analyzer to quantify the amino acids (preceded by hydrolysis for TAA) were included. Studies were excluded if the amino acid contents were determined by microbiological methods. Methods for detection of methionine, cysteine, and tryptophan were evaluated to ensure consistency.

### 2.3. Data Analysis and Standard Unit Conversion

The mean amino acid concentrations in human milk were calculated by averaging the mean values for each included publication. In most studies, total amino acid concentrations were reported in quantities per 100 mL of milk. Free amino acids were usually reported as µmol/L of milk. For data expressed in units per 100 g, the volume-weight correction was ignored. To provide a comparison to the published recommendations, total amino acid concentrations were converted to concentrations per g of total nitrogen while also considering the differences between true and crude protein. The conversion factor of 6.25 was used when applicable. In one study [15], the total nitrogen content was not reported. However, true protein content was detected by the Lowry method [16]. The calculation of total nitrogen was rendered possible by the evidence that only about 80% of the nitrogen in mature human milk is protein nitrogen [17].

Most reports gave means, standard deviations, number of subjects, and number of pooled samples. When “number of subjects” alone was given or values were given for individuals, one breast milk sample was assumed for each subject. For this reason, data were treated as independent in the statistical analysis.

Analysis of Variance (ANOVA) was used to compare the effect of gestational age, lactation stage, and region on TAA and FAA of HM using the SAS^®^ software (version 9.1; SAS Institute, Cary, NC, USA).

## 3. Results

### 3.1. The Dataset

The human milk composition literature reviewed is listed in Table 1, Table 2 and Table 3. We reviewed 83 articles from 18 countries with publication dates ranging from 1941 to 2009 that provided total and/or free amino acid content in human milk for one or more lactation stages in term and/or preterm milk. Based on the inclusion criteria, twenty-six articles providing 79 mean values from 3774 subjects for TAA (Table 1), and 22 studies providing 65 mean values from 4747 subjects for FAA (Table 2) were included in this analysis. Table 3 lists those studies that were excluded from this review. The number of studies was globally unbalanced through all lactation stages and regions. For instance, there were more data from Asia, while there was a paucity of data from Africa. Data on total nitrogen and total amino acid composition of human milk revealed general inter-study consistency, whereas marked variability was seen in the values of the free amino acids between studies.

**Table 1:** Included studies on total amino acid profiles of human milk.

Reference	Region	Country	Term
Atkinson 1980 [18]	NA	Canada	Preterm and Term
Bellomonte 1990 [19]	EU	Italy	Not Specified
Britton 1986 [15]	NA	USA	Preterm and Term
Chavalittamrong 1981 [20]	AP	Thailand	Not Specified
Cheung, Pratt, and Fowler 1953 [21]	NA	Puerto Rico	Not Specified
Darling 1997 and 1999 [22,23]	NA	Canada	Preterm
Darragh 1998 [24]	AP	New Zealand	Term
Davis 1994 [25]	NA	USA	Not Specified
Donovan 1989 [26]	NA	USA	Term
Feng 2009 [27]	EU, AP, NA, SA	9 Countries	Not Specified
Hanning 1992 [28]	NA	Canada	Term
Harzer 1985 [29]	EU	Germany	Term
Janas 1986 [30]	NA	USA	Term
Janas 1987 [31]	NA	USA	Term
Lauber 1979 [32]	AF	Ivory Coast	Not Specified
London Department of Health [33]	EU	England	Term
Lönnerdal 1976 [34]	EU	Sweden	Not Specified
Lönnerdal 1985 [35]	EU	Sweden	Not Specified
Sarwar 1996 [36]	NA	Canada	Preterm and Term
Shaikhiev 1978 [37]	AP	Russia	Not Specified
Svanberg 1977 [6]	AF	Ethiopia and Sweden	Not Specified
Villapando 1998 [38]	NA	USA and Mexico	Term
Wu 2000 [39]	AP	Taiwan	Term
Yamawaki 2005 [40]	AP	Japan	Term
Yonekubo 1989 [41]	AP	Japan	Not Specified
Zhao 1989 [42]	AP	China	Term

**Table 2:** Included studies on free amino acid profiles of human milk.

Reference	Region	Country	Term
Agostoni 2000 [3]	EU	Italy	Term
Agostoni 2000 [7]	EU	Italy	Term
Armstrong 1963 [43]	NA	USA	Not Specified
Atkinson 1980 [18,44]	NA	Canada	Preterm and Term
Carratù 2003 [5]	EU	Italy	Term
Chuang 2005 [8]	AP	Taiwan	Preterm and Term
Darling 1997 and 1999 [22,23]	NA	Canada	Preterm
DeSantiago 1998 [45]	NA	Mexico	Not Specified
Donovan 1989 [26]	NA	USA	Not Specified
Elmastas 2008 [9]	AP	Turkey	Term
Gutikova 2001 [46]	AP	Russia	Not Specified
Harzer 1984 [47]	EU	Germany	Term
Lemons 1983 [2]	NA	USA	Preterm and Term
López-Sánchez Solís 1988 [48]	EU	Spain	Not Specified
Motil 1995 [49]	NA	USA	Term
Pamblanco 1989 [11]	EU	Spain	Preterm and Term
Rassin 1977 [50]	EU and NA	Finland and USA	Not Specified
Singh 2004 [51]	AP	India	Term
Viña 1987 [52]	EU	Spain	Not Specified
Wu 2000 [39]	AP	Taiwan	Term
Wurtman 1979 [53]	NA	USA and Guatemala	Not Specified
Yonekubo 1989 [41]	AP	Japan	Not Specified

**Table 3:** Excluded studies on total and free amino acid profiles of human milk.

Study	Reason for Exclusion
*Total Amino Acids*	
Beach 1941 [54]	Microbiological determination
Block 1946 [55]	Microbiological determination
Davis 1993 [56]	Mean from many studies; Duplicate representation
Davis 1994 [57]	No total nitrogen or total protein reported
DeSantiago 1999 [58]	Marginally nourished lactating women
Flippova 1974 [59]	No method for amino acid quantification
Guo 2007 [60]	Single milk donor
Heine 1991 [61]	Same data as Renner 1983
Järvenpää 1982 [62]	Same data as Rassin 1976
Lemons 1983 [2]	Only protein-bound amino acid profiles excluded
Macy 1949 [63]	Microbiological determination
Macy 1961 [64]	Microbiological determination
Miller 1950 [65]	Microbiological determination
Mitton 1992 [66]	Source of amino acid values not given
Motil 1995 [49]	Only protein-bound amino acid profiles excluded
Nagasawa 1970 [67]	Casein amino acid profile
Nayman 1979 [68]	Mean from many studies; duplicate representation; microbiological determination
Picone 1989 [69]	No information given on milk sampling, number of samples, or method of amino acid quantification
Räihä 2002 [70]	Same data as Nayman 1979
Rassin 1977 [50]	Lactation stage unclear
Renner 1983 [71]	Duplicate representation
Rigo 1994 [72]	Same data as Harzer 1985
Saito 1975 [73]	Microbiological determination; lactation stage unclear
Scott 1990 [74]	No information given on milk sampling or method of amino acid quantification
Soupart 1954 [75]	Lactation stage unclear
Tikanoja 1982 [76]	Lactation stage unclear
Volz 1983 [77]	Same as Agricultural Handbook 1976
Williamson 1944 [78]	Lactation stage unclear
Woodward 1976 [79]	Casein amino acid profile
*Free Amino Acids*	
Farriauz 1971 [80]	Single milk donor
Faus 1984 [81]	Data presented only in chart form
Ghadimi 1963 [82]	Values unconvertible
Nayman 1979 [68]	References by microbiological determination, duplicated representation
Pajarón 1992 [83]	Values represent mean of all stages of lactation
Periago 1994 [84]	Values represent mean of all stages of lactation

### 3.2. Effect of Lactation Stage

The distribution of total and free amino acid concentrations by least-squares means (LS means) according to lactation stage is shown in Table 4 and Table 5, respectively. Total nitrogen and each of the 18 individual total amino acids in human milk sharply declined in the first two months of lactation followed by a considerably slower rate of decrease (Table 4). While concentrations of most individual free amino acids steadily decreased with the progression of lactation, glutamate and glutamine steadily increased after three weeks of lactation, and yielded the greatest level in late mature milk (Table 5). The most abundant was glutamate, with a concentration around 20 times higher than that of other free amino acids, ranging from 960.1 to 1529.0 µmol/L (Table 5). Taurine was also abundant, with an average of 287.1 µmol/L by 2 mo of lactation.

### 3.3. Geographical Distribution

**Figure 1:**
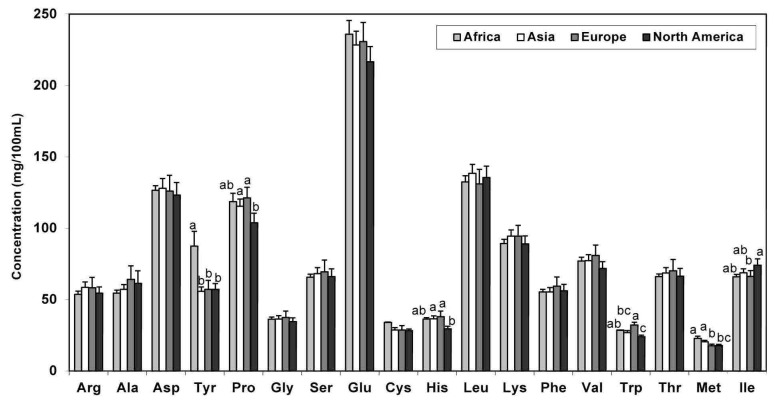
TAA content in human milk according to continent of milk collection (Values are means ± SE). * Means that do not share a common superscript letter are significantly different (*p* < 0.05). ^†^ Includes milk from term and not-specified (NS) groups.

**Figure 2:**
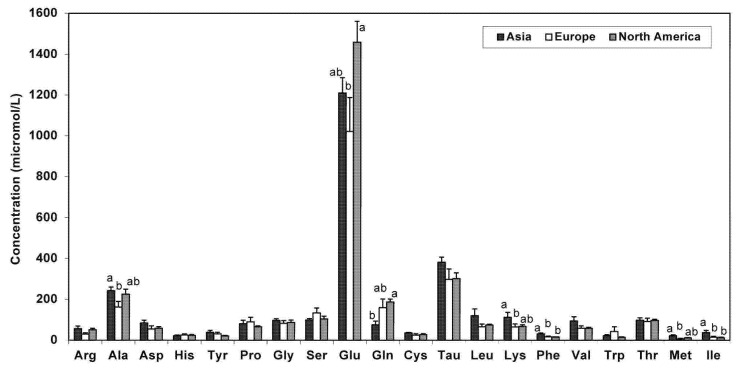
FAA content in human milk according to continent of milk collection (Values are means ± SE). * Means that do not share a common superscript letter are significantly different (*p* < 0.05). ^†^ Includes milk from term and not-specified (NS) groups.

A comparison of the TAA and FAA values among the different geographical continents (Africa, Asia, Europe, and North America) is illustrated in Figure 1 and Figure 2. In regards to TAA, tyrosine appeared to be significantly greater in milk derived from African mothers versus the other regions. On the other hand, levels of proline, histidine, methionine, and tryptophan were lowest in milk derived from North American mothers (Figure 1).

For FAA, there were no data from Africa. Overall, alanine, phenylalanine, methionine, and isoleucine were significantly higher in milk from Asian mothers. Interestingly, glutamate levels were greatest in milk from North America (Figure 2).

### 3.4. Effect of Gestational Age

Only six references were available on preterm total amino acids. These milk samples were collected from 5 to 25 days after birth with an average of 12.5 days postpartum, all from North America. The effect of gestational age was examined by comparing the concentrations of total amino acids and total nitrogen from these six references to that of term transitional milk (Table 6). Results showed that TAA and TN levels were generally higher in preterm human milk than term milk. The concentrations of valine, threonine, and arginine were significantly greater in preterm than term transitional milk. Although preterm milk had higher total nitrogen content than term milk, the difference was not statistically significant.

**Table 4:** Least-square means of human milk total amino acid (TAA) and total nitrogen (TN) content according to lactation stage (Amino acid values in mg/100 mL; TN values in g/L) ^§,^*^,†^.

AA	Lactation Stage				
	Colo	Trans	MT 2 mo	MT4mo	MT18mo
***IAA***					
*His*	57.0 ^a^	38.7 ^b^	29.1 ^c^	26.5 ^c^	24.6 ^c^
*Leu*	206.2 ^a^	147.2 ^b^	118.8 ^c^	104.6 ^d^	94.5 ^d^
*Lys*	141.8 ^a^	99.6 ^b^	82.2 ^c^	68.9 ^d^	66.8 ^d^
*Phe*	95.3 ^a^	59.2 ^b^	46.0 ^c^	39.0 ^d^	43.9 ^cd^
*Val*	125.3 ^a^	79.1 ^b^	67.0 ^c^	58.0 ^d^	54.8 ^d^
*Trp*	43.3 ^a^	30.7 ^b^	24.3 ^c^	20.1 ^d^	21.4 ^cd^
*Thr*	119.3 ^a^	72.3 ^b^	54.9 ^c^	47.2 ^d^	45.6 ^d^
*Met*	28.7 ^a^	21.3 ^b^	17.8 ^c^	16.6 ^cd^	14.3 ^d^
*Ile*	93.5 ^a^	79.5 ^b^	64.7 ^c^	54.8 ^d^	51.6 ^d^
***DAA***					
*Arg*	102.9 ^a^	62.0 ^b^	44.5 ^c^	37.4 ^d^	35.0 ^d^
*Ala*	110.7 ^a^	59.3 ^b^	48.1 ^c^	39.2 ^c^	39.5 ^d^
*Asp*	207.3 ^a^	138.6 ^b^	107.2 ^c^	90.9 ^d^	85.3 ^e^
*Tyr*	100.3 ^a^	69.5 ^b^	52.8 ^c^	50.2 ^c^	49.3 ^c^
*Pro*	165.3 ^a^	125 ^b^	100.3 ^c^	94.9 ^c^	88.3 ^c^
*Gly*	66.2 ^a^	38.1 ^b^	28.9 ^c^	24.3 ^d^	23.5 ^d^
*Ser*	119 ^a^	74.1 ^b^	52.7 ^c^	47.2 ^cd^	44.1 ^d^
*Glu*	332.7 ^a^	241.9 ^b^	201.3 ^c^	189.2 ^cd^	174.4 ^d^
*Cys*	50.2 ^a^	31.0 ^b^	23.5 ^c^	23.9 ^c^	21.2 ^c^
*TN*	**3.5 ^a^**	**2.6 ^b^**	**2.1 ^c^**	**1.9 ^d^**	**1.7 ^d^**

IAA: Indispensable amino acids are those that are essential to the infant [85]. DAA: Dispensable amino acids are those amino acids that are non-essential [85]. ^§^ Colo (0–5 days), Trans (6–20 days), MT2mo (21–58 days), MT4mo (59–135 days), MT18mo (136–540 days). * Means that do not share a common superscript letter are significantly different (*p* < 0.05). ^†^ Includes milk from term and not-specified groups.

**Table 5:** Least-square means of free amino acid (FAA) in human milk according to lactation stage (Values in µmol/L) ^§,^*^,†^.

AA	Lactation Stage							
	Colo	Trans	MT2mo	MT ≥ 2 mo
***IAA***								
*His*	31.7	27.1	21.5	20.9
*Leu*	204.7 ^a^	67.0 ^b^	54.9 ^b^	59.4 ^b^
*Lys*	187.3 ^a^	87.8 ^b^	58.1 ^b^	43.0 ^b^
*Phe*	42.2 ^a^	19.3 ^b^	15.9 ^b^	16.4 ^b^
*Val*	160.1 ^a^	69.2 ^b^	57.4 ^b^	46.3 ^b^
*Thr*	143.1 ^a^	81.6 ^b^	79.1 ^b^	92.9 ^b^
*Met*	34.1 ^a^	18.3 ^b^	10.1 ^bc^	4.0 ^c^
*Ile*	55.1 ^a^	31.6 ^ab^	14.2 ^b^	8.6 ^b^
*Tau*	452.8 ^a^	386.1 ^ab^	287.1 ^bc^	237.9 ^c^
***DAA***								
*Arg*	94.3 ^a^	35.6 ^b^	30.2 ^b^	31.5 ^b^
*Ala*	218.5	189.4	199.9	224.5
*Asp*	114.3 ^a^	60.7 ^b^	55.3 ^b^	58.4 ^b^
*Tyr*	72.4 ^a^	25.8 ^b^	22.3 ^b^	22.1 ^b^
*Pro*	172.0 ^a^	70.5 ^b^	49.1 ^b^	61.7 ^b^
*Gly*	84.6	81.4	84.0	101.4
*Ser*	122.5	80.9	99.8	128.9
*Glu*	1089.9 ^b^	960.1 ^b^	1175.0 ^ab^	1529.0 ^a^
*Gln*	13.5 ^c^	92.6 ^bc^	134.6 ^ab^	225.8 ^a^
*Cys*	27.5	30.5	32.2	28.3

IAA: Indispensable amino acids are those that are essential to the infant [85]. DAA: Dispensable amino acids are those amino acids that are non-essential [85]. ^§^ Colo (0–5 days), Trans (6–20 days), MT2mo (21–60 days); MT > 2 mo (≥61 days). * Means that do not share a common superscript letter are significantly different (*p* < 0.05). ^†^ Includes milk from term, preterm and not-specified groups.

**Figure 3:**
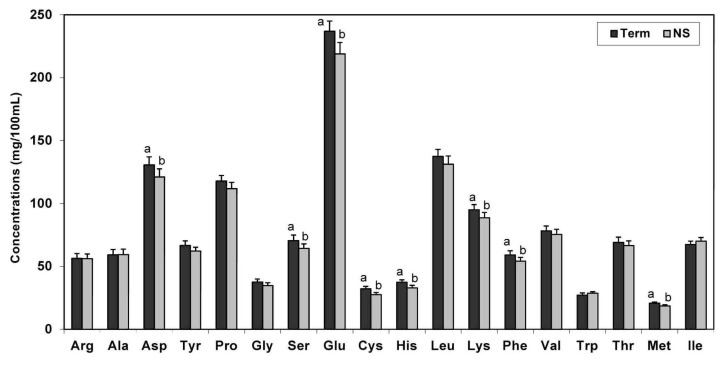
Least-square means of TAA in term human milk compared to not-specified (NS) milk (Values are means ± SE). * Means that do not share a common superscript letter are significantly different (*p* < 0.05).

As not all studies provided a gestational age, the non-specified (NS) and term groups were also compared. Results showed that the indispensable amino acids (IAA) lysine, phenylalanine, histidine, and methionine and the dispensable amino acids (DAA) aspartate, glutamate, serine, and cysteine were higher in term human milk than the NS group (Figure 3).

Concentrations of free amino acids in term milk were compared to preterm human milk, as well as the not-specified (NS) group (Table 7). Overall, the levels of FAA were similar between term and preterm milk, with the exception of glutamine, which was significantly lower by nearly one half in preterm than term human milk (Table 7).

### 3.5. Comparison to Global Human Milk Standards

Results of the indispensable and dispensable TAA data from term human milk in this systematic review are remarkably similar to the recommendations of the Scientific Committee on Food (SCF) 2003 (European Commission, 2003) and ESPGHAN 2005/CODEX 2007 [86], with the exception of phenylalanine being lower in our data set (Table 8). Conversely, the values of the Life Sciences Research Office (LSRO), 1998 [1], and the World Health Organization (WHO), 2007 [87], are considerably lower than the concentrations obtained from this data set.

**Table 6:** Least-square means of TAA and TN in transitional term milk compared to transitional preterm milk (Amino acid values in mg/100 mL; TN values in g/L) *^,§^.

*AA*	Gestation Period	
	Preterm	Term
***IAA***		
*His*	41.7	34.5
*Leu*	192.4	159.3
*Lys*	134.7	107.8
*Phe*	79.1	63.8
*Val*	117.1 ^a^	83.3 ^b^
*Trp*	32.1	21.4
*Thr*	102.1 ^a^	68.4 ^b^
*Met*	27.8	21.1
*Ile*	95.5	84.0
***DAA***		
*Arg* ^†^	93.8 ^a^	64.9 ^b^
*Ala*	90.9	56.8
*Asp*	174.4	130.1
*Tyr* ^†^	88.3	68.8
*Pro*	153.0	128.2
*Gly* ^†^	55.0	41.7
*Ser*	111.8	90.2
*Glu*	305.3	252.9
*Cys* ^†^	32.0	28.0
*TN*	*3.5*	*2.8*

IAA: Indispensable amino acids are those that are essential to the infant [85]. DAA: Dispensable amino acids are those amino acids that are non-essential [85]. * Means that do not share a common superscript letter are significantly different (*p* < 0.05). ^†^ Cysteine, taurine, tyrosine, arginine and glycine may be considered conditionally essential amino acids for the preterm infant [13]. ^§^ Transitional milk defined as 6–20 days lactation.

**Table 7:** Least-square means of FAA in term milk compared to preterm human milk and not-specified (NS) group (Values in µmol/L) *.

AA	Gestation Periods		
	Preterm	Term	NS
***IAA***			
*His*	19.8	28.6	27.4
*Leu*	80.6 ^ab^	65.0 ^b^	143.9 ^a^
*Lys*	104.0	76.0	102.2
*Phe*	21.1	23.9	25.4
*Val*	79.9	73.0	96.9
*Thr*	82.9	92.6	121.9
*Met*	17.1	16.1	16.7
*Ile*	23.0	32.7	26.5
*Tau* ^†^	366.7 ^ab^	387.4 ^a^	268.8 ^b^
***DAA***			
*Arg* ^†^	56.7	36.7	50.2
*Ala*	181.7	210.9	231.6
*Asp*	67.8	77.9	70.9
*Tyr* ^†^	33.1	34.9	38.9
*Pro*	92.4	68.3	104.3
*Gly*	81.5	99.3	82.8
*Ser*	76.0	125.0	123.0
*Glu*	1201.2	1168.1	1196.2
*Gln*	52.9 ^b^	159.9 ^a^	137.0 ^ab^
*Cys* ^†^	21.9	31.7	35.2

IAA: Indispensable amino acids are those that are essential to the infant [85]. DAA: Dispensable amino acids are those amino acids that are non-essential [85]. * Means that do not share a common superscript letter are significantly different (*p* < 0.05). ^†^ Cysteine, taurine, tyrosine, arginine and glycine may be considered conditionally essential amino acids for the preterm infant [13].

**Table 8:** Comparison of present analysis to global TAA standards (Values in mg/g TN) ^†^.

	Present Analysis	Koletzko 2005; CODEX 2007 [86,88]	LSRO 1998 [1]	Panel of Macronutrients 2005; Kleinman 2009 [89,90]	USDA 2009 [91]	European Commission 2003 [92]	WHO/FAO 2007 [87]
No. of Studies	26	7	4	4	*	6	3
***IAA***							
*His*	143 ± 2.8	141	*	112.13	136.72	137.5	102.38
*Leu*	570 ± 8.1	586	491.4	492.38	566.41	575	468
*Lys*	389 ± 6.0	395	355.39	336.38	410.16	393.5	336.38
*Phe*	224 ± 6.2	282	181.84	195.0	273.44	287.5	204.75
*Val*	318 ± 6.0	315	258.38	273.0	371.09	306.25	268.13
*Trp*	111 ± 2.8	114	75.56	87.75	97.66	112.5	82.88
*Thr*	263 ± 3.4	268	220.35	229.13	273.44	268.75	214.5
*Met*	86 ± 1.8	85	71.66	78.0	117.19	81.25	78
*Ile*	305 ± 5.0	319	251.55	277.83	332.03	312.5	268.13
***DAA***							
*Arg*	211 ± 3.2	196	*	*	253.91	*	112.13
*Ala*	221 ± 4.2	*	*	*	214.84	*	185.25
*Asp*	503 ± 6.7	*	*	*	488.28	*	*
*Tyr*	242 ± 8.1	259	199.88	229.13	312.5	262.5	253.5
*Pro*	498 ± 9.0	*	*	*	488.28	*	390
*Gly*	135 ± 2.0	*	*	*	156.25	*	112.13
*Ser*	256 ± 9.0	*	*	*	253.91	*	243.75
*Glu*	994 ± 14.7	*	*	*	1015.63	*	*
*Cys*	117 ± 2.9	131	94.09	107.25	117.19	131.25	82.88
Asp + Asn							438.75
Glu + Gln							867.75

IAA: Indispensable amino acids are those that are essential to the infant [85]. DAA: Dispensable amino acids are those amino acids that are non-essential [85]. ^†^ Includes milk from term and not-specified (NS) groups. * Data not available.

## 4. Discussion

The complete characterization and quantitation of protein and non-protein nitrogen in human milk serves as an appropriate nutritional guide for understanding and defining an infant’s protein and amino acid requirements. The amino acid content of human milk is comprised of TAA and FAA, and FAA make up a significant component of NPN, ~8%–22% in human milk [4,5]. FAA enter into the circulation sooner after ingestion than protein-derived amino acids, and their rapid absorption contributes significantly to the initial changes in FAA levels in the infant’s plasma [3,11,93]. FAA, however, are typically overlooked in the scientific literature focusing on protein composition. Therefore, our objective was to compile a comprehensive database characterizing both the TAA and FAA content of human milk to better understand the quantitative and qualitative changes in amino acid composition through lactation.

To this end, we collected data, published as early as 1941 to 2010, from more than 70 studies to ascertain if gestational age, lactation stage, and geographical region significantly influence levels of total and free amino acids in preterm and term human milk. Overall, we observed that TAA (and TN) values were relatively consistent among studies; however, the effects of lactation stage, gestational age, and geographical origin still need to be considered, since these appear to influence the amino acid profiles and TN values.

Results from this study confirm that a majority of the variation in amino acid composition of human milk is caused by stage of lactation. The greatest decline in TAA concentration occurs over the first 4 mo of lactation with levels remaining relatively stable thereafter (Table 4). This correlates to the changing protein needs of the growing infant [94]. Interestingly, some FAA (alanine, glycine, serine, glutamine, and glutamate) increase with progressing lactation. For instance, glutamine was nearly 20 times higher in mature milk than its lowest value in colostrum (Table 5). Glutamate, known as the most abundant FAA, has many beneficial functions to the growing infant by providing ketoglutaric acid for the citric acid cycle, possibly acting as a neurotransmitter in the brain, and serving as a major energy substrate for intestinal cells [3]. Recently, it was suggested that the high levels of free glutamate in breast milk and extensively hydrolyzed formula are responsible for the lower daily intakes of these diets [95]. It has also been proposed that very low-birth weight infants receiving glutamine supplementation have less tissue catabolism and enhanced gluconeogenesis [96]. Once again, the full physiological importance of all the FAA to infant growth and development has yet to be established [12,50].

It has often been reported that the amino acid pattern and overall protein concentration of preterm milk are generally similar to those of term milk [44,97]. On an absolute basis, our comparison of preterm and term milk showed that TN and all AA were higher in concentration in preterm than in term milk (Table 6), which is consistent with other studies that reported significantly higher concentrations of protein [36,98] and total nitrogen [99] in preterm milk than term human milk. These results indicate that preterm milk may be a more appropriate source of protein and certain amino acids than term milk to accommodate for the rapid growth rates of premature infants. However, our study focused only on a few studies from the transitional stage of lactation in preterm milk, and significant differences were only observed for valine, threonine, and arginine; whereas, amino acid patterns through lactation were not addressed in this study (Table 6).

There have been discussions as to whether the concentration and proportion of NPN in preterm human milk is significantly different from that of term milk [2,44,100,101,102]. Similarly, the influences of gestational age on the level of individual free amino acids remain uncertain. The concentrations of most FAA from this analysis were similar in term and preterm milk (Table 7). Our analysis, however, is limited to a handful of available published studies on preterm milk. These data emphasize the need to carefully consider not only the total protein needs but also the essential and non-essential amino acid requirements of preterm infants. These differences in preterm and term human milk also reiterate the need for more research associated with longer lactation periods, especially given the increased survival rates of preterm infants born at earlier gestational ages.

Significant differences among the different geographical regions were observed for a handful of TAA (tyrosine, proline, histidine, methionine, and tryptophan) and FAA (lysine, phenylalanine, methionine, and isoleucine). Results from Feng *et al*. (2009) showed total amino acid profiles were similar across nine different countries [27], agreeing with the present study. Some research indicates that maternal diet quality may influence TAA and FAA in mothers’ milk [53,103], yet in other studies protein content is well preserved in mothers consuming protein deficient diets [104,105]. Although we excluded data from mothers consuming experimental diets, we did not exclude data from mothers whose typical diets may have had lower protein quality or inadequate intake. However, a more recent study showed no relation between the amino acid concentration in maternal diet and milk of women in Northern China [106].

In contrast to the TAA data, the values for FAA showed large inter-study variability. Previously published data on the concentration of FAA in human milk show wide variations, depending on corresponding stages of lactation [6,50,53]. For example, Carratù *et al*. observed differences in median and mean values after analysis of 195 samples in the first month of lactation, despite careful standardization of collection procedures to minimize variation in FAA values [5]. The differences between median and mean values indicated an abnormal distribution of data, especially for those amino acids present in traces, which may be a characteristic of human milk [5].

## 5. Conclusions

This systematic review offers a thorough characterization of human milk total and free amino acid patterns throughout the course of lactation and across several geographical areas. It represents a useful dataset for the evaluation of protein quality and quantity of breast milk substitutes for preterm and term infants. Its comprehensive nature may also serve as a guide to support future efforts in the establishment and revision of global and/or regional human milk amino acid reference values throughout the first year of life.

## Abbreviations

HM: human milk
TN: total nitrogen
TAA: total amino acids
FAA: free amino acids
IEG: International Expert Group
NPN: Non-protein nitrogen
AP: Asia
EU: Europe
NA: North America
AF: Africa
ANOVA: Analysis of Variance
IAA: Indispensable amino acids
DAA: Dispensable amino acids
NS: Not-specified
PC: Principal component
Mo: month
Wk: week
Yr: year
H: hour
d: day
g: gram
LS: least-squares
Leu: Leucine
Lys: Lysine
Phe: Phenylalanine
Val: Valine
Trp: Tryptophan
Thr: Threonine
Met: Methionine
Ile: Isoleucine
Tau: Taurine
Arg: Arginine
Ala: Alanine
Asp: Aspartate
Tyr: Tyrosine
Pro: Proline
Gly: Glycine
Ser: Serine
Glu: Glutamate
Gln: Glutamine
Cys: Cysteine
Colo: Colostrum
Trans: Transitional
MT: Mature
LacStage: Lactation Stage
